# The Effectiveness of Peer Education, Mentorship and Role‐Playing Approaches in Developing Clinical Skills Among Iranian Nursing Students: A Systematic Review

**DOI:** 10.1002/hsr2.71665

**Published:** 2025-12-30

**Authors:** Asma Ghonchehpour, Mina Alipoor, Zakieh Farmitani, Fereshteh Ghorbani, Esmat Nouhi

**Affiliations:** ^1^ Nursing Research Center Kerman University of Medical Sciences Kerman Iran; ^2^ Department of Anesthesia, Faculty of Nursing and Paramedicine Rafsanjan University of Medical Sciences Rafsanjan Iran; ^3^ Razi Faculty of Nursing and Midwifery Kerman University ofMedical Sciences Kerman Iran

**Keywords:** mentorship, peer education, role play, systematic review

## Abstract

**Background and Aims:**

This study aimed to assess the effectiveness of peer education, mentorship, and role play approaches in developing clinical skills in nursing students.

**Methods:**

We conducted a systematic review using specific criteria to identify randomized controlled trials and quasi‐experimental focused on nursing students. From the period 2000 to November 28, 2024, six databases, namely Web of Science, Scopus, PubMed, EMBASE, Cochrane, and Google Scholar, were searched through search strategy and the use of Boolean operators.

**Results:**

After screening 1821 titles and abstracts, 22 full texts were reviewed, and 6 studies were included in the final analysis. The findings revealed that mentorship significantly improved nursing students' clinical competencies compared to other methods. Role‐playing effectively enhanced practical skills such as CPR and triage. Peer education fostered collaboration and confidence but demonstrated inconsistent results in enhancing clinical skills.

**Conclusion:**

Mentorship is the most effective method for improving clinical skills, while role‐playing excels in practical training. Peer education supports collaboration with inconsistent results. Integrating these methods can enhance nursing education. Further research is needed for broader validation.

## 1 Introduction

Clinical education, which makes up nearly half of the nursing curriculum, enables students to integrate theoretical knowledge with practical skills in real clinical settings. Its purpose is to build professional competence, critical thinking, and the ability to provide safe, evidence‐based care. Nursing schools must therefore train graduates who can meet patients' needs with current knowledge and strong clinical skills [1]. Effective clinical education depends largely on selecting appropriate teaching methods [2], as traditional teacher‐centered approaches often fail to actively engage students in meaningful learning [3, 4, 5]. Therefore, innovative student‐centered strategies such as peer learning, mentorship, and role‐playing have been increasingly recommended in nursing education.

Peer education is an effective teaching approach for nursing students [6]. In this approach, near peers, senior students, or peers at the same academic or experiential level help others in learning, understanding, acquiring, and retaining skills, forming attitudes, and applying knowledge [7]. When peer assessment strategies are adopted in the teaching‐learning process, numerous benefits arise, including fostering motivation and responsibility for learning, providing constructive feedback, enhancing critical thinking, developing deep subject knowledge, and promoting mutual understanding among peers [8, 9].

In mentorship, learners work alongside an experienced mentor to acquire skills and techniques. Mentorship is described as a process where a mentor guides another individual (mentee) in developing skills and knowledge for professional growth [10, 11, 12]. Utilizing experienced clinical nurses as mentors for nursing students is a novel educational practice that opens new horizons in clinical learning [13]. Mentorship is a supportive, collaborative, and voluntary relationship (not hierarchical) where the mentor serves as a model for the learner [14]. It improves self‐efficacy, clinical confidence, and academic achievement for both mentors and mentees while benefiting educational institutions [14, 15, 16].

Role‐playing is an educational method that allows learners to actively participate in simulated scenarios to enhance learning and skill development [17]. It provides learners with the opportunity to acquire specific skills, such as problem‐solving, stress and crisis management, time management, risk‐taking, and critical thinking. By analyzing case studies in role‐playing scenarios, learners gain self‐awareness and can evaluate their knowledge, skill gaps, and professional growth [18].

Despite increasing interest in active learning strategies in nursing education, current evidence in Iran remains fragmented and method‐specific, with no comprehensive synthesis comparing different approaches. Previous reviews have either examined single educational methods or generalized findings from international contexts, which may not be applicable to Iran due to cultural and educational differences in clinical training. Therefore, a systematic comparison of peer education, mentorship, and role‐playing within the Iranian nursing context is essential to identify the most effective teaching strategies for improving clinical competence and to provide evidence‐based guidance for curriculum development and policy reform.

The relevance of peer education, mentorship, and role‐playing in nursing education is grounded in contemporary learning theories and their demonstrated impact on clinical competence. Peer education is based on Bandura's Social Learning Theory, which emphasizes observational learning and self‐efficacy development through interaction with peers. It enhances communication, teamwork, and confidence in clinical practice [19, 20]. Mentorship, rooted in Vygotsky's Zone of Proximal Development, provides individualized guidance and feedback from experienced mentors, fostering professional identity formation and clinical decision‐making [21]. Role‐playing aligns with Kolb's Experiential Learning Theory, enabling students to apply theoretical knowledge to simulated clinical scenarios and improve critical thinking and problem‐solving skills in a safe environment [22]. Therefore, these three strategies are pedagogically complementary and collectively contribute to the development of clinical competence, justifying their inclusion in this review.

## 2 Aims

This systematic review aims to evaluate the effectiveness of peer education, mentorship, and role‐playing in developing the clinical skills of Iranian nursing students.

## 3 Literature Review

Educational effectiveness refers to the process of acquiring diverse skills that enhance individuals' knowledge [23]. One of the main concerns of educational systems and policymakers is ensuring the effectiveness of teaching methods. Bahar et. al (2022) indicated that the peer education model in nursing education has been shown to diminish learning anxiety and to positively influence the acquisition of psychomotor skills [24]. Wachira (2019) studied the perceptions of mentorship practices among nursing students in Kenya, finding that mentorship improved students' confidence and positively contributed to their professional growth [25]. Rezaei Fard et al. (2020) compared clinical wound dressing skills among nursing students taught through mentorship, faculty‐led instruction, and peer learning in Iran. While the mentorship group demonstrated the best performance among all groups, the researchers recommended employing both mentorship and peer learning methods to develop clinical skills [26]. Teo et. al (2024) demonstrated that the similarity between mentorship and complex adaptive systems, emphasizing the dynamic, emergent, and nonlinear characteristics of mentoring relationships, and advocates for a shift in paradigm toward more supportive and efficient mentorship practices in medical education [12].

However, no systematic review to date has simultaneously compared the effectiveness of peer education, mentorship, and role‐playing in developing clinical skills among Iranian nursing students. Previous reviews have examined these methods separately, but a direct comparison is still lacking. Therefore, this study addresses this gap by synthesizing available interventional evidence and identifying the most effective approaches for clinical education.

## 4 Materials and Methods

The PRISMA 2020 approach for Systematic Review (PRISMA‐SR) was used to conduct this study [27].

### 4.1 Eligibility Criteria

In this systematic review, we applied specific inclusion and exclusion criteria to identify relevant studies and analyze their results. This systematic review aims to examine the effectiveness of peer education, mentorship, and role play methods on developing clinical skills in Iranian nursing students. Educational methods can be based on the content of the subject, which are divided into two categories: traditional methods and non‐traditional (new) methods. Traditional methods include: lecture method, group discussion, one‐to‐one training, practical demonstration and re‐practical demonstration and new methods such as: gamification, simulation, role playing, role modeling, reverse education, electronic education and peer education, mentorship [28]. The decision to include only peer education, mentorship, and role‐playing in this systematic review was based on several key considerations. First, these approaches are among the most widely studied and evidence‐based active learning strategies in Iranian nursing education, demonstrating significant potential to improve clinical skills. Second, initial literature searches revealed that these three methods have a substantial number of high‐quality randomized controlled trials and quasi‐experimental studies, ensuring a robust and reliable analysis. Third, these methods specifically target different aspects of clinical skill development—peer education promotes collaboration and confidence, mentorship enhances overall competency through guidance, and role‐playing provides practical, hands‐on experience—making them highly relevant and complementary in evaluating effective teaching strategies. Lastly, focusing on these three approaches allows for a more targeted and in‐depth comparison, providing clearer insights and actionable recommendations for educators The clinical performance assessment tools for medical students include: Structured Observation and Clinical Assessment (SOAP), Nursing Student Competency Instrument (CINS), Nursing Competency Questionnaire (CNCQ), OSCE, logbook, direct observation of performance (DOPS), and Mini clinical evaluation exercise (Mini‐ CEX) [29]. Improvement of clinical competence was defined as measurable enhancement in practical clinical performance, psychomotor skills, or clinical decision‐making abilities, assessed using validated tools such as OSCE, PPCS‐R, DOPS, or structured skill checklists.

### 4.2 Inclusion Criteria

a. **Study design**: randomized controlled trials, and quasi‐experimental studies.
b. **Participants:** All articles must have been conducted in nursing students in Iran.
c. **Intervention:** Any educational intervention such as peer education, mentorship and role play that leads to the acquisition or improvement of clinical competence in nursing students.
d. **Comparison**: studies with any type of intervention as a comparison in nursing students.
e. **Outcome:** clinical skills such as Perioperative Competency, vital sign control (Blood pressure, Pulse, Breathing, Temperature), IV injection, Intramuscular injection, Subcutaneous injection, Wound dressing, Catheterization, ESI triage, CPR, wound care, oral medication administration, heat application, Cold application, glucose check, physical examination skills [30, 31, 32, 33, 34, 35, 36].
f. **Publication Type:** Our inclusion criteria encompassed original articles and articles in press.
g. **Language and Publication Date:** We limited the language criterion to English and excluded articles published before 2000.

### 4.3 Exclusion Criteria

a. **Study Design:** studies conducted with other designs, such as observational studies, reviews, cross sectional study, conference proceedings, letters, editorials, cohort, qualitative studies, and case reports or series.
b. **Population:** studies that conducted on the other students of medical sciences and Countries other than Iran.
c. **Intervention:** studies assessing interventions without a clear description or implementation details.
d. **Comparison:** studies lacking proper control groups or those with insufficient methodological quality were excluded to ensure the reliability of the findings.
e. **Outcome:** Studies that do not provide a clear and measurable outcome related to the acquisition or improvement of clinical competence in nursing students were excluded.

### 4.4 Information Sources

We extensively searched across electronic databases, including Embase, PubMed, Google Scholar, Scopus, Cochrane Library and Web of Science [37], until 28 November 2024. Furthermore, we expanded our search by reviewing the reference lists of the studies included in our analysis to ensure a comprehensive exploration of pertinent literature.

### 4.5 Search Strategy

we expanded our search by reviewing the reference lists of the studies included in our analysis to ensure a comprehensive exploration of pertinent literature.

The keywords used included: Mentor”, “Mentorships”, “Mentorship”, “Coaching”, “Mentoring”, “Mentors”, “Playing”, “Role Play”, “Role Playing”, “Role Plays”, “Peer Learning”, “Peer Education”, “Peer Support”, “Peer Mentoring”, “Peer Counseling”, “Peer Teaching”, “Clinical Competency”, “Clinical Competencies”, “Competencies, Clinical”, “Competency, Clinical”, “Competence, Clinical”, “Clinical Skill”, “Clinical Skills”, “Skill, Clinical”, “Skills, Clinical” “Nursing Student”, “Nursing Students”, “Student, Nursing”, “Nurses”, “Pupil”, “Nurse”, “Pupil Nurse”, “Pupil Nurses (Supporting Information: file1).

### 4.6 Selection Process

**Figure 1:**
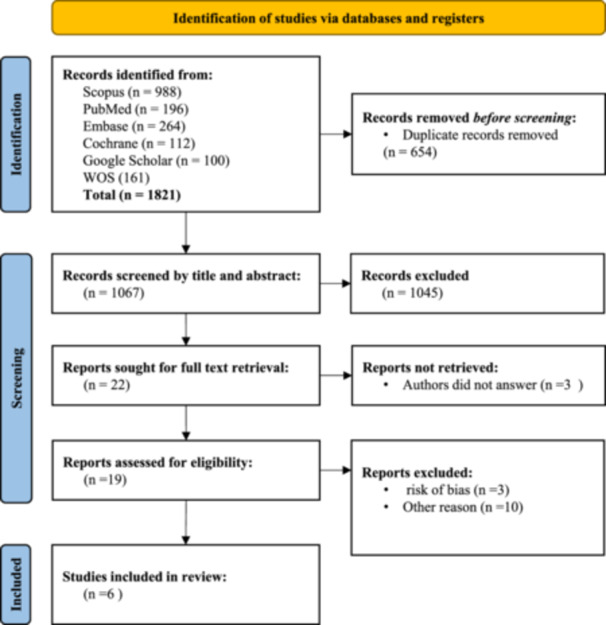
Flowchart of screening process.

At the outset, one of the researchers transferred all search results from the databases into EndNoe Desktop software, removing any duplicates. Following this, two researchers independently evaluated the titles and abstracts of the articles according to established eligibility criteria. If there were any disagreements regarding study selection, a comprehensive review of the full text was performed, with a third researcher involved if needed. Efforts were made to obtain inaccessible articles and unpublished data by contacting the corresponding authors of the eligible studies. The screening process was visually represented using the PRISMA 2020 flow diagram [38] (Figure 1).

### 4.7 Data Collection Process

Two authors independently extracted data from all articles. Any differences were addressed through discussions that included a third author. The form included key information such as author(s), year, country, objective, type of study, inclusion/exclusion criteria, Clinical skills tool, Education method, sample size, intervention group, comparison/control group, outcome.

### 4.8 Study Risk of Bias Assessment

In order to evaluate the quality of the studies, we employed the CASP Randomized Controlled To assess the quality of the studies, we utilized the CASP Randomized Controlled Trial Standard Checklist, which consists of 11 questions divided into four sections: (A) evaluating the validity of the core study design, (B) examining the strength of the study methodology, (C) analyzing the reporting of results, and (D) considering the local implications of the findings [39]. Studies that failed to meet more than half of the checklist criteria or were ambiguous were excluded. Additionally, we performed a duplicate evaluation of the risk of bias for each study using the Revised Cochrane risk‐of‐bias tool for randomized trials (RoB 2). This tool identifies potential sources of bias across five areas [1]: bias from the randomization process [2], bias from deviations from intended interventions [3], bias from missing outcome data [4], bias in outcome measurement, and [5] bias in the selection of reported results [38]. The included articles which fully met the above criteria were defined as grade A, suggesting that the possibility of various biases was low. Articles satisfying the above quality standards were defined as grade B, indicating that the possibility of bias was moderate. Original articles which did not meet the above quality standards at all were defined as grade C, indicating that the possibility of bias was high [39].

### 4.9 Publication Bias

We assessed publication bias by employing funnel plots and the Egger's test. Funnel plots provide a visual representation of the effect estimates of studies plotted against their standard errors. In the absence of publication bias, the funnel plot should exhibit a symmetrical inverted funnel shape. We visually examined the funnel plots to identify any signs of asymmetry and used the Egger's test to quantitatively evaluate the presence of publication bias. A p‐value below 0.10 was considered as significant publication bias. If publication bias was identified, our plan was to employ trim‐and‐fill analysis to account for its influence on the overall effect estimate.

## 5 Results

### 5.1 Literature Search

Out of the initial 1821 articles identified through database searching, 654 duplicates were removed. The remaining 1067 titles and abstracts were screened, yielding 22 full‐text articles for review. Three articles lacked full text, and ten articles were excluded for other reasons. Nine articles were subsequently assessed for quality and risk of bias. Ultimately, six articles contributed to the study inclusion (Figure 1; Table 1).

**Table 1:** Publication bias.

Article	Q1	Q2	Q3	Q4	Q5	Q6	Q7	Q8	Q9	Q10	Q11
Neda Mirbagher Ajorpaz et al., 2016 [30]	Yes	No	Yes	No	Yes	Yes	Yes	Yes	Yes	Yes	No
Dehghan et al., (2018) [31]	Yes	Yes	Yes	No	Yes	Yes	Yes	Yes	Yes	Yes	Yes
Delnavaz et al., (2018) [32]	Yes	Yes	Yes	Yes	Yes	Yes	Yes	Yes	Yes	Yes	Yes
Nasr‐Esfahani et al., (2019) [33]	Yes	Yes	Yes	No	Yes	Yes	Yes	Yes	Yes	Yes	Yes
Rezaei Fard et al., (2020) [34]	Yes	Yes	Yes	No	Yes	Yes	Yes	Yes	Yes	Yes	Yes
Nasiri et al., (2023) [36]	Yes	Yes	Yes	Yes	Yes	Yes	Yes	Yes	Yes	Yes	Yes

Q1: Did the study address a clearly focused research question?

Q2: Was the assignment of participants to interventions randomized?

Q3: Were all participants who entered the study accounted for at its conclusion?

Q4: Were the participants ‘blind’ to intervention they were given? AND Were the investigators ‘blind’ to the intervention they were giving to participants? AND Were the people assessing/analyzing outcome/s ‘blinded.’

Q5: Were the study groups similar at the start of the randomized controlled trial?

Q6: Apart from the experimental intervention, did each study group receive the same level of care?

Q7: Were the effects of intervention reported comprehensively?

Q8: Was the precision of the estimate of the intervention or treatment effect reported?

Q9: Do the benefits of the experimental intervention outweigh the harms and costs?

Q10: Can the results be applied to your local population/in your context?

Q11: Would the experimental intervention provide greater value to the people in your care than any of the existing interventions?

### 5.2 Study Characteristics

The studies included in this review were conducted in Iran between 2016 and 2023 (Table 2).

**Table 2:** The results of the seven studies.

Author(s), Year	Country	Objective	Type of study	Inclusion/Exclusion criteria	Education method	Sample size	Clinical skills tool	Intervention group	Control group	Outcome
Neda Mirbagher Ajorpaz et al., 2016	Iran	Assess mentoring effects on perioperative competence	A single‐blind clinical trial	Inclusion: Inclusion criteria for participants were final‐semester bachelor's degree‐seeking students interning at the Shahid Beheshti Hospital Exclusion: Exclusion criteria included being a guest student and being absent for more than two sessions during the two months of the study	Mentorship program	60 OR nirsing students	Persian Perceived Perioperative Competence Scale (The PPCS‐R)	two months, the experimental group attended in the OR with similar conditions as the control group. Assigning mentors for the experimental group was the only difference between the two group's conditions in the OR	In the first two months, the control group attended the OR five days a week for their routine clinical practice	In the experimental group, the difference between the mean scores of clinical competence before (19.43 ± 2.80) and after (27.86 ± 1.87) the intervention was significant (*p* ≤ 0.001). After intervention, the difference between the mean scores of the control (3.9 ± 0.15) and experimental (8.61 ± 0.68) groups was significant (*p* = 0.003).
Dehghan et al., 2020	Iran	Evaluate integrated training on nursing education	interventional study	Inclusion: Nursing students in clinical training; Exclusion: N/A	Integrated training (OSCE)	58 nursing students	Clinical lab tests, OSCE	peer training (26 subjects). In peer learning, a strong student would be paired with a poor student and a strong student was a peer trainer	routine training (32 subjects). The apprenticeship of the nursing principles and techniques unit included 12 sessions in 12 days from 7:30 am to 12:00 am	Scores of all the students were increased significantly at the end of the semester, but in terms of the total score of the clinical skills (14.79 ± 1.52 vs. 18.52 ± 0.84), the difference was insignificant (*p* = 0.29).
Samira Delnavaz et al., 2018	Iran	Compare lecture vs role‐playing for triage education	Experimental study	Inclusion: who had completed the “nursing crisis course” and had no experience of working in emergency departments signed the informed consent form and participated in the present study Exclusion: Students with experience of working in emergency departments	Lecture vs role‐playing	50 nursing students	The knowledge and practice of all the students in the two groups were assessed after one month in the form of post‐test by using a questionnaire which was developed by the researchers based on the latest edition of ESI triage	In the role‐playing group, the students were subjected to the scenarios using role‐playing in which the other firstsemester nursing students played the role of the patients in the scenario, and they were trained to play the role accurately.	In the lecture group, scenarios adapted from the fourth edition of the guideline on ESI (Gilboy et al., 2012) were presented by lecture and using PowerPoint for 3 h.	The mean knowledge in both groups, (lecture = 23.60 ± 5.21 vs role‐playing= 36.24 ± 3.32) improved significantly (*p* < 0.001). The post‐test score showed a significant difference in practice scores between the two groups, and the mean score was higher in the role‐playing group (23.08 ± 2.69). compared with that of the lecture group (17.64 ± 4.73) (*p* < 0.001).
Mohammad Nasr‐Esfahani et al., 2019	Iran	Assess role‐playing method for CPR training	Semi‐experimental	Inclusion: Nursing students without clinical CPR experience; Exclusion: Students with prior CPR certification	Role‐playing	70 students	Advanced CPR performance checklist	Role‐playing method	Traditional method	The paired t‐test showed a significant difference between the mean performance scores after intervention between Role‐playing and control groups, respectively (*p* = 0.01). Furthermore, independent t‐test showed that a significant improvement in CPR performance for the role‐playing group compared with control (post‐intervention difference; *p* = 0.010).
Zeynab Rezaei Fard et al., 2020	Iran	Compare mentorshi, peer, and faculty‐led learning for wound care	Experimental 3‐group study	Inclusion: First and second‐year nursing students; Exclusion: Students not enrolled in wound care training	peer‐led learnin, mentorship‐led, faculty‐led control group	102 students	researcher‐made checklist which included 21 items	For the PL group peer educators, two senior nursing students with excellent Grade Point Averages (GPAs) (GPA 17 or more of 20 in the Iranian educational system) and who were competent in clinical skills, were selected. They were provided with training on surgical dressings and wound care using Taylor's Clinical Nursing Skills book regarding clinical principles and clinical nursing skills. For ML group, two surgical personnel were selected who were qualified registered nurses had at least two years of clinical education experience and their clinical and educational skills were confirmed by educational and clinical supervisors. For the FL control group, instruction was carried out by faculty as per normal. In this group nursing students received training from two faculty educators who were qualified and experienced in nursing. These faculty educators are traditional type educators who are teaching students now in for many years, are qualified to BS standard but are not familiar with new educational methods	In the control group, the education was conducted by faculty educators. The practical element for all groups was carried out on male and female surgical wards at the patients’ bedside	Based on findings, after the intervention, the mean (SD) scores of surgical dressing and wound care skills were 28.24 (4.63), 31.76 (4.89), and 29.12 (5.33) for the peer‐led, mentor‐led and faculty‐led groups, respectively. There was no significant difference between mentor group and faculty group or between peer group and faculty group (*p* > 0.05). However, the findings did demonstrate statistical difference in performance in surgical dressings and wound care techniques in the mentorship group method compared to the peer education method (*p* = 0.006).
Mohammad‐Amin Nasiri et al., 2023	Iran	Compare mentorship vs educational videos for physical examination skills	This study was a clinical audit with three group pretest posttest design.	Inclusion: Nursing students; Exclusion: Exclusion criteria included missing one or more educational sessions, and not watching the educational video according to the specified schedule (in the educational video group) and the allowance to withdraw from the study whenever they want	Mentorship, educational videos	64 nursing student	32‐item checklist for assessing the students’ skills in examining the respiratory system (10 items), cardiovascular system (13 items), and 12 cranial nerves (9 items).	**Mentor group:** Selected students from a master's program in medical‐surgical nursing were trained to become mentors. They received five 2‐h sessions on mentoring principles, and after the pretest, they taught physical examination (PE) skills in a clinical setting for three consecutive weeks. The mentor taught students on real patients, providing hands‐on experience and correcting mistakes in real‐time. **Educational video group:** Students in this group received educational videos on PE skills for respiratory, cardiovascular, and nervous systems through Telegram messenger. The videos were watched twice weekly, and students learned PE skills through self‐study. They did not have the opportunity for hands‐on practice like the mentor group.	**Control group:** This group received standard educational content without any special intervention. They attended three 1‐h lecture sessions on each system and performed PE skills in the clinical setting under the supervision of staff and faculty members, following the regular curriculum.	At baseline, students in all groups scored less than half of the possible scores in all three systems, and the mean scores of the three groups (educational video, Mentorship, Control) were not statistically different (*p* = 0.798). After the intervention, the mean scores of students in the mentorship group increased significantly in all three systems (*p* < 0.001), whereas the mean scores of students in the educational video group and the control group did not change significantly (*p* > 0.05). Furthermore, after the intervention, the mean scores of the educational video group and the control group did not significantly differ in any of the three systems (*p* > 0.05). The ANCOVA showed that with posttest score as the covariate, PE skills in all three systems improved one week after the intervention in the mentor group compared to the control group and the educational video group (P = O. OO1). However, PE skills in all three systems did not improve one week after the intervention in the educational video group compared to the control group (*p* > 0.05).

### 5.3 Peer Education

Study characteristics Peer learning was investigated in two studies conducted in Iran in 2020 and 2022, with sample sizes of 58 and 102 students, respectively.

### 5.4 Peer Education Interventions

In a study by Dehghan et al. (2020) [31], students of varying skill levels were paired for peer learning. In a study by Zeynab Rezaei Fard et al. (2020) [34], senior students with high GPAs mentored junior students.

### 5.5 Peer Education Outcomes

In a study by Dehghan et al. (2020), scores improved in the peer‐learning group but did not differ significantly from the control group. In a study by Rezaei Fard et al. (2020), the peer‐learning group performed similarly to the faculty‐led group but was outperformed by the mentorship group.

### 5.6 Mentorship

Study characteristics Studies on mentorship were conducted in Iran between 2016 and 2023 and comprised three studies with sample sizes ranging from 60 to 102 participants. These studies examined the impact of mentorship programs on the clinical competencies of nursing students, using tools such as the Persian Perceived Perioperative Competence Scale (PPCS‐R) and researcher‐developed checklists for evaluation.

### 5.7 Mentorship Interventions

In a study by Mirbagher Ajorpaz et al. (2016) [30], students in the intervention group were mentored during their clinical practice, while the control group followed routine clinical training. Rezaei Fard et al. (2020) [34], compared three approaches: mentorship by experienced nurses, peer education, and faculty‐led traditional instruction; the mentorship group received guidance from professional nurses with at least 2 years of clinical education experience. In a study by Nasiri et al. (2023) [36], mentors were master's‐level nursing students who provided hands‐on training in clinical settings.

### 5.8 Mentorship Outcomes

In a study by Mirbagher Ajorpaz et al. (2016), clinical‐competence scores in the mentorship group improved significantly (*p* ≤ 0.001). In a study by Rezaei Fard et al. (2020), the mentorship group outperformed the peer‐learning group (*p* = 0.006), but there was no significant difference compared with the faculty‐led group. In a study by Nasiri et al. (2023), mentorship significantly improved scores across all evaluated systems (*p* < 0.001).

### 5.9 Role‐Playing

Study characteristics Role‐playing interventions were examined in two studies conducted in Iran in 2018 and 2019, including 50 and 70 nursing students, respectively, comparing role‐playing with traditional methods (lectures or standard instruction) for teaching clinical skills such as triage and CPR.

### 5.10 Role‐Playing Interventions

In a study by Delnavaz et al. (2018) [32], students in the intervention group engaged in role‐playing scenarios in which they acted as patients and nurses, while the control group received lecture‐based training using PowerPoint. In a study by Nasr‐Esfahani et al. (2019) [33], the intervention group practiced CPR in a simulated environment, whereas the control group received traditional training.

### 5.11 Role‐Playing Outcomes

In a study by Delnavaz et al. (2018), both groups showed improvements in knowledge and skills, but the role‐playing group scored significantly higher than the lecture group (*p* < 0.05). In a study by Nasr‐Esfahani et al. (2019), the role‐playing group demonstrated significantly better performance than the control group (*p* = 0.01).

Overall, these findings suggest that educational strategies that incorporate structured supervision, real‐time feedback, and active learner engagement—such as mentorship and role‐playing—are more effective in developing clinical competence than peer‐led learning alone.

## 6 Discussion

The primary aim of this systematic review was to investigate various interventions aimed at improving clinical skills in nursing students. The study specifically examined randomized controlled trials (RCTs) and quasi‐experimental that evaluated interventions such as peer education, mentorship, and role playing. The goal was to assess their efficacy in improving clinical skills.

In the reviewed studies, educational interventions focused on clinical practices of nursing students such as Perioperative Competency, vital sign control (Blood pressure, Pulse, Breathing, Temperature), IV injection, Intramuscular injection, Subcutaneous injection, Wound dressing, Catheterization, ESI triage, CPR, wound care, oral medication administration, heat application, Cold application, glucose check, physical examination skills.

These findings suggest that educational effectiveness is not determined solely by the type of teaching strategy, but by the level of structure, guidance, and feedback embedded within each method. Mentorship demonstrated stronger and more consistent effects because it incorporates expert supervision and individualized feedback, which are essential for developing complex psychomotor and clinical reasoning skills. In contrast, peer education provides emotional and social support but may lack the instructional precision required for mastering advanced clinical procedures if not properly structured. Role‐playing, while effective for practicing emergency decision‐making and teamwork, may be limited by the artificial nature of simulated scenarios and variability in facilitator skill.

The educational context in Iran may contribute to these findings. Limited faculty training in modern pedagogical strategies, unequal access to simulation facilities, and hierarchical clinical environments may reduce the effectiveness of interactive learning strategies.

According to Kolb's experiential learning theory, clinical competence develops through cycles of guided practice and reflection. Mentorship facilitates this process by allowing learners to observe expert role models and receive formative feedback in real clinical environments [21, 22]. Peer education aligns with Bandura's social learning theory but its outcomes depend heavily on peer competency and instructional design. Without mentor oversight, misconceptions may be unintentionally reinforced among peers [40]. Role‐playing supports active learning based on constructivist principles, but its impact depends on the realism of clinical scenarios and structured debriefing [17].

The results of this study showed that peer education fosters a supportive environment but does not significantly improve clinical skills compared to other methods. The limited effectiveness of this approach may stem from the absence of expert guidance, as students rely on each other's knowledge and skills, which may vary widely in quality [31, 34]. These outcomes align with Bandura's social learning theory, which emphasizes the importance of observation and interaction in building self‐efficacy and reducing anxiety. However, the lack of structure and consistency may limit the method's effectiveness in developing complex clinical skills. Peer education is more suitable for fostering communication, collaboration, and confidence among students rather than teaching advanced clinical techniques [41, 42]. Mixed results in peer education studies may be due to variations in sample size and peer selection criteria. Studies with untrained peer educators or small cohorts showed weaker outcomes [31], consistent with Secomb's findings that peer learning requires structured preparation to be effective [40]. Bahar et. al (2022) indicated that the peer education model in nursing education has been shown to diminish learning anxiety and to positively influence the acquisition of psychomotor skills [24].

The results demonstrated that mentorship significantly enhances clinical competencies [30, 36] Mentorship provides personalized feedback and step‐by‐step guidance, which helps students improve their skills through direct, hands‐on training.

The effectiveness of mentorship can be attributed to Kolb's experiential learning theory, which highlights the role of concrete experience and reflective observation in the learning process. By working with an experienced mentor, students gain valuable insights and develop a deeper understanding of clinical practices. Additionally, mentorship boosts confidence and motivation by providing a secure and supportive environment [11, 13].

The superior outcomes associated with mentorship may be attributed to its reliance on guided reflective practice and expert scaffolding, which enhance clinical reasoning and skill acquisition [21, 22]. Teo et al. (2024) show similarities between mentorship and complex adaptive systems, highlighting the dynamic, emergent, and nonlinear nature of mentoring relationships and efficient in medical education [12].

The results also showed that role‐playing significantly improved practical skills such as CPR and triage compared to traditional methods Studies by Delnavaz et al. (2018) [32] and Nasr‐Esfahani et al. (2019) [33]. Role‐playing enables students to simulate real‐life scenarios, fostering critical thinking and decision‐making.

Role‐playing resonates with constructivist learning theory, which emphasizes active engagement and real‐world problem‐solving. By immersing students in realistic scenarios, this method bridges the gap between theoretical knowledge and practical application. It also provides a safe environment for students to experiment, make mistakes, and learn without the pressure of real‐life consequences [17, 18].

A blended approach that combines mentorship and role‐playing may help maximize the strengths of both strategies. In this model, mentors supervise role‐playing sessions and guide students through structured clinical scenarios, allowing them to practice decision‐making, teamwork, and technical procedures in a safe environment. After each scenario, mentors provide individualized feedback and facilitate reflective discussions, reinforcing correct techniques and correcting errors. This approach aligns with Kolb's Experiential Learning Theory by linking simulation‐based practice with expert‐guided reflectio. Therefore, integrating role‐playing under mentor supervision may be a practical teaching strategy for nursing programs, especially in resource‐limited settings where simulation laboratories are available but underutilized due to lack of instructional support.

Previous international research supports the findings of this review and highlights the global relevance of active learning strategies in nursing education. For example, Burgess et al. (2018) emphasized that mentorship enhances professional identity formation and clinical competence among health profession students in Australia, similar to the outcomes reported in Iranian studies [21]. Likewise, Secomb (2008) showed in a systematic review that peer education improves confidence and collaborative learning in clinical settings across multiple countries, although its effect on technical clinical skills varies [40]. Additionally, role‐playing has been reported to enhance clinical decision‐making and communication skills internationally [17]. These findings suggest that integrating structured mentorship with peer‐assisted learning and simulation‐based strategies can create more effective and transferable educational frameworks. Policymakers and nursing educators are encouraged to adopt blended educational models, provide mentor training programs, and allocate institutional resources to support evidence‐based teaching methods that improve clinical competence and student outcomes.

## 7 Study Limitations

While this review offers valuable insights, several limitations should be acknowledged:

Studies were conducted in Iran, limiting the generalizability of findings to other cultural and educational contexts. Variations in sample sizes, interventions, and evaluation tools across studies complicate direct comparisons and synthesis of results. Also, most studies assessed outcomes immediately after interventions, making it difficult to evaluate the long‐term impact of these educational methods on clinical competence. Furthermore, the studies examined a limited range of clinical skills, leaving gaps in understanding the effectiveness of these methods for other essential nursing competencies.

### 7.1 Future Research Recommendations

Future studies should go beyond short‐term assessments and explore the long‐term retention of clinical skills through longitudinal study designs. In addition, mixed‐method approaches are recommended to better capture not only measurable performance outcomes but also students' learning experiences, confidence, and professional identity formation. Future trials should also evaluate broader clinical outcomes such as readiness for professional practice, clinical decision‐making ability, teamwork, and patient safety indicators. Comparative studies across different educational contexts and resource settings are also needed to determine the generalizability and scalability of peer education, mentorship, and role‐playing interventions in nursing education.

### 7.2 Practical Implications

Based on these conclusions, implement mentorship as a core component of nursing education to boost clinical competencies. Combine mentorship with experiential learning activities (e.g., role‐play) to augment practical skills beyond traditional methods. Encourage peer collaboration as a supplementary approach, but monitor its impact on performance consistency. Support further research with diverse, longitudinal studies and mixed‐method designs to link educational strategies with patient safety and clinical judgment outcomes.

### 7.3 Practical Implications for Educators and Policymakers

The results of this review suggest several practical implications for nursing education. First, mentorship should be integrated as a structured component of clinical training, as it consistently demonstrated significant positive effects on clinical competence. Educational policymakers should support the implementation of formal mentorship programs by allocating institutional resources, providing mentor training workshops, and recognizing mentorship in faculty promotion criteria. Second, peer education can be used as a supplementary strategy to enhance collaboration and confidence among students, especially in large clinical groups where instructor supervision is limited. Third, role‐playing should be combined with simulation‐based learning to improve decision‐making, teamwork, and emergency response skills in realistic clinical scenarios. Finally, national nursing education authorities should develop clear guidelines that encourage blended learning models, combining mentorship, peer learning, and simulation to strengthen clinical competency development.

## 8 Conclusion

This systematic review demonstrates that mentorship significantly enhances clinical competencies among nursing students, outperforming other educational methods. While peer education offers collaborative benefits, it lacks consistent performance improvements. Role‐playing proves effective in improving practical skills compared to traditional teaching methods. Overall, integrating mentorship and experiential learning strategies can create a more impactful educational environment, effectively preparing nursing students for their professional roles. It is crucial to conduct additional research, including larger and more diverse studies, to establish more definitive results and provide guidance for clinical practice. Future research should include longitudinal designs to assess skill retention, mixed‐method approaches to measure learners' confidence and professional readiness, and outcome metrics such as patient safety and clinical judgment accuracy.

## Author Contributions

**Asma Ghonchehpour:** conceptualization, data curation, formal analysis, writing – original draft, writing – review and editing, validation, methodology. **Mina Alipoor:** data curation, writing – original draft, writing – review and editing. **Zakieh Farmitani:** data curation, writing – original draft, writing – review and editing. **Fereshteh Ghorbani:** Formal analysis, data curation, writing – original draft, writing – review and editing. **Esmat Nouhi:** writing – original draft, writing – review and editing, formal analysis, visualization, data curation, supervision, methodology, conceptualization.

## Funding

The authors received no specific funding for this work.

## Consent

The authors have nothing to report.

## Conflicts of Interest

The authors declare no conflicts of interest.

## Transparency Statement

The lead author Esmat Nouhi affirms that this manuscript is an honest, accurate, and transparent account of the study being reported; that no important aspects of the study have been omitted; and that any discrepancies from the study as planned (and, if relevant, registered) have been explained.

## Supporting information

STERATEGY.

## Data Availability

The data are available upon request to the corresponding author after signing appropriate documents in line with ethical application and the decision of the Ethics Committee.
