# Supplementary material for: The Effectiveness of Peer Education, Mentorship and Role‐Playing Approaches in Developing Clinical Skills Among Iranian Nursing Students: A Systematic Review
Source: Health Sci Rep. 2025 Dec 30;9(1):e71665. doi: 10.1002/hsr2.71665 (PMC12754267; doi:10.1002/hsr2.71665)
Supplement: Supplementary file 1 — STERATEGY. [file HSR2-9-e71665-s001.docx]

| Data base | strategy | number |
| --- | --- | --- |
| PubMed | ("Mentor" OR "Mentorships" OR "Mentorship" OR "Coaching" OR "Mentoring" OR "Mentors" OR "Playing" OR "Role Play" OR "Role Playing" OR "Role Plays" OR "Peer Learning" OR "Peer Education" OR "Peer Support" OR "Peer Mentoring" OR "Peer Counseling" OR "Peer Teaching") AND ("Clinical Competency" OR "Clinical Competencies" OR "Competencies, Clinical" OR "Competency, Clinical" OR "Competence, Clinical" OR "Clinical Skill" OR "Clinical Skills" OR "Skill, Clinical" OR "Skills, Clinical") AND ("Nursing Student" OR "Nursing Students" OR "Student, Nursing" OR "Nurses" OR "Pupil" OR "Nurse" OR "Pupil Nurse" OR "Pupil Nurses") AND ("1999"[Date - Publication] : "2026"[Date - Publication]) | 196 |
| WOS | TS=("Mentor" OR "Mentorships" OR "Mentorship" OR "Coaching" OR "Mentoring" OR "Mentors" OR "Playing" OR "Role Play" OR "Role Playing" OR "Role Plays" OR "Peer Learning" OR "Peer Education" OR "Peer Support" OR "Peer Mentoring" OR "Peer Counseling" OR "Peer Teaching") AND TS=("Clinical Competency" OR "Clinical Competencies" OR "Competencies, Clinical" OR "Competency, Clinical" OR "Competence, Clinical" OR "Clinical Skill" OR "Clinical Skills" OR "Skill, Clinical" OR "Skills, Clinical") AND TS=("Nursing Student" OR "Nursing Students" OR "Student, Nursing" OR "Nurses" OR "Pupil" OR "Nurse" OR "Pupil Nurse" OR "Pupil Nurses") AND PY=(1999-2026) | 161 |
| SCOPUS | TITLE-ABS ( "Mentor" OR "Mentorships" OR "Mentorship" OR "Coaching" OR "Mentoring" OR "Mentors" OR "Playing" OR "Role Play" OR "Role Playing" OR "Role Plays" OR "Peer Learning" OR "Peer Education" OR "Peer Support" OR "Peer Mentoring" OR "Peer Counseling" OR "Peer Teaching" ) AND ( "Clinical Competency" OR "Clinical Competencies" OR "Competencies, Clinical" OR "Competency, Clinical" OR "Competence, Clinical" OR "Clinical Skill" OR "Clinical Skills" OR "Skill, Clinical" OR "Skills, Clinical" ) AND ( "Nursing Student" OR "Nursing Students" OR "Student, Nursing" OR "Nurses" OR "Pupil" OR "Nurse" OR "Pupil Nurse" OR "Pupil Nurses" ) AND PUBYEAR > 1999 AND PUBYEAR < 2026 | 988 |
| Cochrane Library | ("Mentor" OR "Mentorships" OR "Mentorship" OR "Coaching" OR "Mentoring" OR "Mentors" OR "Playing" OR "Role Play" OR "Role Playing" OR "Role Plays" OR "Peer Learning" OR "Peer Education" OR "Peer Support" OR "Peer Mentoring" OR "Peer Counseling" OR "Peer Teaching") AND ("Clinical Competency" OR "Clinical Competencies" OR "Competencies, Clinical" OR "Competency, Clinical" OR "Competence, Clinical" OR "Clinical Skill" OR "Clinical Skills" OR "Skill, Clinical" OR "Skills, Clinical") AND ("Nursing Student" OR "Nursing Students" OR "Student, Nursing" OR "Nurses" OR "Pupil" OR "Nurse" OR "Pupil Nurse" OR "Pupil Nurses") AND Publication Year Range: 1999-2026 | 112 |
| EMBASE | ('Mentor' OR 'Mentorships' OR 'Mentorship' OR 'Coaching' OR 'Mentoring' OR 'Mentors' OR 'Playing' OR 'Role Play' OR 'Role Playing' OR 'Role Plays' OR 'Peer Learning' OR 'Peer Education' OR 'Peer Support' OR 'Peer Mentoring' OR 'Peer Counseling' OR 'Peer Teaching') AND ('Clinical Competency' OR 'Clinical Competencies' OR 'Competencies, Clinical' OR 'Competency, Clinical' OR 'Competence, Clinical' OR 'Clinical Skill' OR 'Clinical Skills' OR 'Skill, Clinical' OR 'Skills, Clinical') AND ('Nursing Student' OR 'Nursing Students' OR 'Student, Nursing' OR 'Nurses' OR 'Pupil' OR 'Nurse' OR 'Pupil Nurse' OR 'Pupil Nurses') AND [1999-2026]/PY | 264 |
